# Insecticide resistance status and mechanisms in *Aedes aegypti* populations from Senegal

**DOI:** 10.1371/journal.pntd.0009393

**Published:** 2021-05-10

**Authors:** Ndeye Marie Sene, Konstantinos Mavridis, El Hadji Ndiaye, Cheikh Tidiane Diagne, Alioune Gaye, El Hadji Malick Ngom, Yamar Ba, Diawo Diallo, John Vontas, Ibrahima Dia, Mawlouth Diallo

**Affiliations:** 1 Medical Zoology Pole, Institut Pasteur de Dakar, Dakar, Sénégal; 2 Institute of Molecular Biology and Biotechnology, Foundation for Research and Technology Crete, Greece; 3 MIVEGEC (Infectious Diseases and Vector: Ecology, Genetics, Evolution and Control), IRD (Institut de recherché pour le Développement), Montpellier, France; 4 Department of Crop Science, Agricultural University of Athens, Athens, Greece; Centers for Disease Control and Prevention, UNITED STATES

## Abstract

*Aedes aegypti* is the main epidemic vector of arboviruses in Africa. In Senegal, control activities are mainly limited to mitigation of epidemics, with limited information available for *Ae*. *aegypti* populations. A better understanding of the current *Ae*. *aegypti* susceptibility status to various insecticides and relevant resistance mechanisms involved is needed for the implementation of effective vector control strategies. The present study focuses on the detection of insecticide resistance and reveals the related mechanisms in *Ae*. *aegypti* populations from Senegal.

Bioassays were performed on *Ae*. *aegypti* adults from nine Senegalese localities (Matam, Louga, Barkedji, Ziguinchor, Mbour, Fatick, Dakar, Kédougou and Touba). Mosquitoes were exposed to four classes of insecticides using the standard WHO protocols. Resistance mechanisms were investigated by genotyping for pyrethroid target site resistance mutations (V1016G, V1016I, F1534C and S989P) and measuring gene expression levels of key detoxification genes (*CYP6BB2*, *CYP9J26*, *CYP9J28*, *CYP9J32*, *CYP9M6*, *CCEae3a* and *GSTD4*).

All collected populations were resistant to DDT and carbamates except for the ones in Matam (Northern region). Resistance to permethrin was uniformly detected in mosquitoes from all areas. Except for Barkédji and Touba, all populations were characterized by a susceptibility to 0.75% Permethrin. Susceptibility to type II pyrethroids was detected only in the Southern regions (Kédougou and Ziguinchor). All mosquito populations were susceptible to 5% Malathion, but only Kédougou and Matam mosquitoes were susceptible to 0.8% Malathion. All populations were resistant to 0.05% Pirimiphos-methyl, whereas those from Louga, Mbour and Barkédji, also exhibited resistance to 1% Fenitrothion. None of the known target site pyrethroid resistance mutations was present in the mosquito samples included in the genotyping analysis (performed in > 1500 samples). In contrast, a remarkably high (20-70-fold) overexpression of major detoxification genes was observed, suggesting that insecticide resistance is mostly mediated through metabolic mechanisms. These data provide important evidence to support dengue vector control in Senegal.

## Author summary

In Senegal, as in most African countries, the arbovirus epidemics control policy relies on the control of the main vector *Ae*. *aegypti* though insecticide applications. Vector control strategies have been largely adopted without information on the vector populations’ insecticide resistance mechanisms. We profiled here the resistance status of nine *Ae*. *aegypti* populations from Senegal to four classes of insecticides and their related mechanisms. Our findings revealed high resistance to carbamates, a relative susceptibility of southern populations to pyrethroids and a variable efficacy of organophosphates. Resistance to pyrethroids was driven by a significant overexpression of detoxification genes linked to insecticide metabolism. Our results contribute towards a more targeted and efficient control of *Ae*. *aegypti* populations and thus of arbovirus epidemics in Senegal.

## Introduction

In recent years, infectious diseases, particularly arboviral ones, are appearing more frequently with epidemics occurring throughout Africa [1]. Yellow fever (YF), despite the availability of an effective vaccine, has re-emerged in 2016–2017 in Angola and Nigeria [2]. In Senegal, the virus was recently detected in 2015 in Kédougou, an area considered crucial for sylvatic YF virus circulation [3]. Zika (ZIK), which caused devastating epidemics with grave clinical manifestations (e.g., Guillain-Barré syndrome, microcephaly) in 2013 in the Pacific Islands and then in America, is endemic in Africa and Southeast Asia [4]. Among all arboviruses isolated from mosquitoes in south-eastern Senegal, Zika virus (ZIKV) has the highest annual amplification frequency [5]. According to the World Health Organization (WHO), 3.9 billion people, in 128 countries, are at risk of infection with dengue viruses. In Africa, dengue is widespread throughout the tropics, with local variations in risk influenced by rainfall, temperature and rapid urbanization [6]. Recent years have been marked by several dengue outbreaks in major cities of Senegal, such as Touba, Louga, Fatick, Mbour and Dakar [7–9]. Among the vectors of these arboviruses, *Ae*. *aegypti* represented by two forms in Africa (*Ae*. *ae*. *aegypti* that evolved from *Ae*. *ae*. *formosus* the ancestral wild type form) and *Ae*. *albopictus* are the most adapted to the human environment. They thrive in both urban and suburban areas [10] and are therefore the most suspected in causing epidemics. In Senegal, *Ae*. *aegypti* is predominantly present [11].

Since there is no specific treatment and efficient vaccine available for most of the diseases transmitted by *Ae*. *aegypti*, vector control with the use of insecticides, remains the only available method to efficiently to confront outbreaks [12]. Unfortunately, most of the vector control strategies are facing operational challenges with the emergence and development of insecticide resistance sustained by different mechanisms [13]. Contrary to Asia and South America, which have long integrated the permanent surveillance and control of *Ae*. *aegypti* and *Ae*. *albopictus* in their arbovirus control strategies, in Africa, vector control of these species is limited to reactive campaigns in response to epidemics [14]. In Senegal, there is no vector control program targeting *Aedes aegypti*. Usually propoxur is used for spatial spaying during outbreaks. In addition, data are limited on the susceptibility status of *Ae*. *aegypti* with only one study carried out, in the locality of Dakar, following a dengue outbreak in 2009 [15]. This study reported that *Ae*. *aegypti* were highly resistant to DDT, susceptible to 1% fenitrothion and 0.75% permethrin, and showed reduced susceptibility to 0.05% deltamethrin, 0.05% lambda-cyhalothrin and 1% propoxur. The vector control programs existing in Africa target primarily *Anopheles* vectors and are based predominately on indoor residual spraying (IRS) and insecticide treated nets (ITN) [16,17]. Among the four main classes of insecticides registered for public health (pyrethroids, carbamates, organophosphates, organochlorines), pyrethroids are predominantly used [10,18]. Their intensive use in both vector control and agriculture has induced insecticide resistance in *Anopheles* species [19]. However, these effects have not been well documented in *Aedes* mosquitoes that are known to have different ecology and behavior. Knowledge on *Aedes* insecticides resistance and the relevant mechanisms involved remains limited in Africa [20].

The mechanisms for insecticide resistance are complex and include behavioral and physiological alterations in mosquitoes [1]. The voltage-gated sodium channel (VGSC) target site of *Ae*. *aegypti* is the most studied insecticide resistance mechanism. Numerous VGSC knockdown resistance (kdr) mutations have been identified in *Ae*. *aegypti* worldwide (V1016I, V410L, S989P, I1011V, V1016G, I1011M and F1534C). Among these mutations, three variants (V410L,V1016I and F1534C) have been detected in Africa [21,22]. Metabolic resistance occurs through increased insecticide metabolism or sequestration by detoxification enzymes. Several genes of the P450 family, especially from the CYP9 and CYP6 subfamilies, carboxy/choline esterases (CCEs) and glutathion-S-transferases (GSTs) have been strongly associated with insecticide resistance in *Aedes* mosquitoes [21]. To our knowledge, apart from one study performed in Burkina Faso, no other report on *Ae*. *aegypti* detoxification genes expression exists for other West African countries [23,24].

The re-emergence of dengue epidemics calls for an effective vector control program. Monitoring mosquito resistance to insecticides is essential; however, relevant information is currently lacking in Senegal. Thus, we carried out the present study in nine Senegalese localities in order to characterize the insecticide resistance status of *Ae*. *aegypti* and to investigate its underlying mechanisms.

## Materials and methods

### Ethics statement

Ethical approval was not required for this study. The study did not involve humans and related data or experiment on neither endangered or protected animal species. The study protocol was carefully explained to the chief and inhabitants of each village investigated to obtain their informed oral consent. Informed oral consent was also obtained from the heads of each household in which mosquito samples were collected.

### Study sites

**Fig 1:**
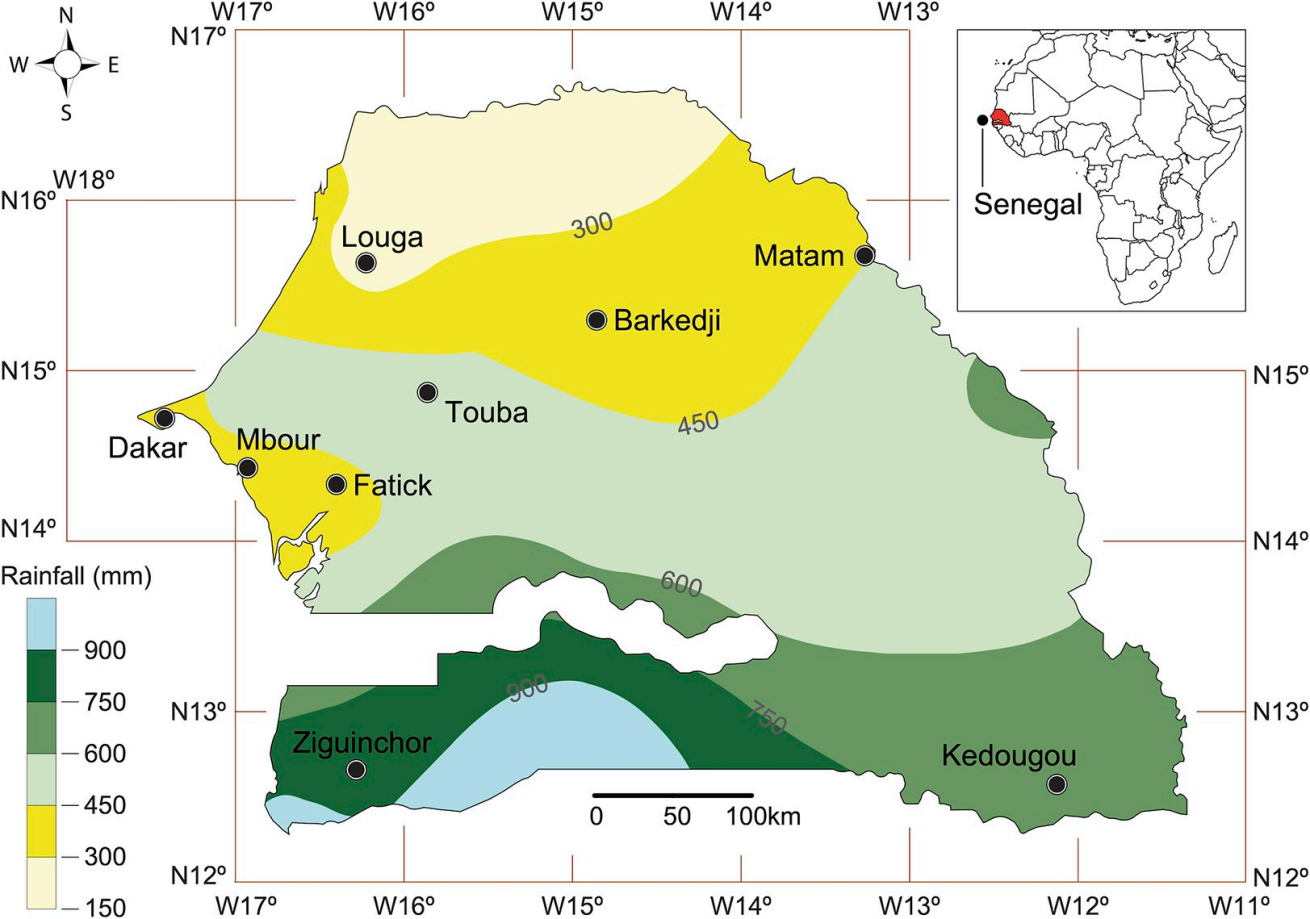
Map showing the nine sites where *Ae*. *aegypti* mosquitoes were collected and the different bioclimatic zones. This map was created using the R software (version 4.0.2) and the package rgdal using an empty shapefile from the HDX website (https://data.humdata.org/dataset/senegal-administrative-boundaries) available under Creative Commons Attribution 4.0 International licence.

*Ae*. *aegypti* mosquitoes were collected from nine Senegalese localities (Dakar, Touba, Fatick, Mbour, Louga, Barkédji, Matam, Ziguinchor and Kédougou) from August 2017 to December 2019 (Fig 1). Except, for localities of Touba and Fatick, where the mosquitoes were collected between October and December 2019, during a dengue epidemic, collections were performed between August 2017 and March 2018 in the 7 other locations.

The localities sampling locations were selected based on the differences in their ecological characteristics, human population density and occurrence of previous arbovirus epidemics. Senegal is characterized by a tropical climate, with a dry season from November to May and a rainy season from June to October. In 2018, the population of Senegal was estimated at 15,726,037 people within an area of ​​196,722 km^2^. There are strong bio-geographical disparities between the 14 regions of the country. Average annual rainfall follows an increasing gradient of 300 mm to 1200 mm from North to South of the country. This distribution of rainfall determines three main climatic zones: a forest zone in the south, a savannah with trees in the center and a semidesert zone in the north (S1 Table).

### Collection and rearing of mosquitoes

Immature forms of *Ae*. *aegypti* mosquitoes (eggs, larvae and pupae) were collected during the rainy season between August 2017 to December 2019 in the domestic and peri-domestic environments of each of the nine localities. Larvae and pupae were collected from potential *Ae*. *aegypti* breeding sites (tires, discarded containers, barrels, etc.) in all localities, except Mbour were where eggs were collected using ovitraps, and were then transported to the Medical Zoology Pole of Institut Pasteur de Dakar. After collection, all immature forms were reared to adults. Emerging adults were identified using an appropriate key [25] and *Ae*. *aegypti* females were blood-fed using guinea pigs to obtain F1 eggs. F1 eggs were then reared to the adult stage and F1 females were maintained in standard conditions (10% Sucrose, 28°C temperature and 70–80% relative humidity). The *Aedes aegypti* New Orleans, Cayman, and Rockefeller laboratory strains were generously provided by the Liverpool Insect Testing Establishment (LITE) and the Swiss Tropical and Public Health Institute (Swiss TPH) to be used as controls, in the molecular assays.

### Insecticide bioassays

Bioassays were carried out using standard WHO tests kits for adult mosquitoes [26]. The Vector Control Research Unit, School of Biological Sciences (Universiti Sains Malaysia), a WHO collaborating Centre, provided us with insecticide-impregnated papers. The following insecticides were used: pyrethroids (0.25% and 0.75% permethrin), 0.05% deltamethrin, 0.03% and 0.5% lambda-cyhalothrin, 0.05% alpha-cypermethrin), organochlorines (4% DDT), carbamates (0.1% propoxur, 0.1% bendiocarb), organophosphates (0.8% and 5% malathion, 0.05% pirimiphos-methyl and 1% fenitrothion). Tests were performed on 3–5 days old unfed F1 females. Batches of 20–25 females were exposed to insecticide-impregnated papers for 1 hour. For each test, two batches of 20–25 females were exposed to untreated papers as control. The numbers of mosquitoes used for each insecticide and their controls are presented in the supplementary S2 Table. The numbers of knocked-down female mosquitoes were recorded after 10, 15, 20, 30, 40, 50 and 60 min of exposure to pyrethroids or DDT. After 1h exposure, female mosquitoes were again transferred into observation tubes, fed with 10% sucrose and maintained at 27–28°C temperature and 70–80% relative humidity. The number of dead female mosquitoes was recorded 24h after exposure.

## Detection of resistance mechanisms

### DNA extraction

Mosquito gDNA was extracted using CTAB 2% [27] from susceptible, pyrethroid- and DDT- resistant mosquitoes. For each insecticide tested, either all available mosquitoes or a maximum of 24 randomly selected individuals were used for genotyping. Each mosquito was homogenized using a sterile pestle in 200μl of CTAB 2%. The lysate was incubated at 65°C for 5 min. After adding 200 μl of chloroform, the mixture was mixed by inversion, kept on ice for 3 min and then centrifuged for 5 min at 12 000 x g, at room temperature. The supernatant was collected in a fresh tube in which 200 μl of isopropanol were added. The resulting mixture was centrifuged for 15 min at 12,000 x g and the supernatant was discarded. The DNA pellet was washed by adding 200 μl of 70% ethanol. After centrifugation for 5 min at 20,000 x g, the pellet was dried in a speed-vac and 50 μl of sterile pure water were added. Samples were then stored at -20°C until processing.

### Species identification

The molecular identification of *Ae*. *aegypti* species was performed using a TaqMan assay as previously published by Kothera et al [28].

### Genotyping of *kdr* V1016G, V1016I, S989P and F1534C mutations

Allele-specific PCR (AS-PCR) using previously published protocols for the V1016G [29] and the F1534C [30] mutations was used. The modifications included differences in the final volume and the quantity of genomic DNA used.

For V1016G *kdr* genotyping, each reaction was performed in a 10 μl volume reaction containing 1.5 mM MgCl_2_, 1X PCR buffer, 0.2 mM dNTPs, 0.5 μM of the forward primer G1016F: (5′-ACCGACAAATTGTTTCCC-3), 0.125 μM of each specific reverse primer Gly1016R (5′-GCGGGCAGGGCGGCGGGGGCGGGGCCAGCAAGGCTAAGAAAAGGTTAACTC-3’) and Val1016R (5′ GCGGGCAGCAAGGCTAAGAAAAGGTTAATTA-3’), 0.2 μM Taq polymerase and 1μl gDNA. The amplification consisted of an initial denaturation step of 94°C for 2 min, followed by 35 cycles of 30 sec at 94°C (denaturation), 30 sec at 55°C (annealing), and 30 sec at 72°C (extension) and a final extension step at 72°C for 2 min. Genotyping of the F1534C *kdr* mutation was carried out in 10 μl reactions containing 1.5 mM MgCl_2_, 1X PCR buffer, 0.2 mM dNTPs, 1 μM Taq polymerase and 1μl genomic DNA. The following primers was used: 0.5 μM forward primer Phe (5′-GCGGGCTCTACTTTGTGTTCTTCATCATATT-3), 0.165 μM reverse primer Cys (5′GCGGGCAGGGCGGCGGGGGCGCGGGGCCTCTACTTTGTGTTCTTCATCATGTG-3’) and 0.5 μM of the common primer 5′-TCTGCTCGTTGAA GTTGTCGAT-3 ’. PCR products were mixed after amplification with 3 μl of 6X Loading Dye and run on either a 4% (V1016G) or 3% (F1534C) agarose gel and stained with ethidium bromide solution for UV visualization. For the V1016G mutation, the sizes of amplified products were 60 bp for the wild-type alleles and 80 bp for mutant ones whereas for the F1534C mutation, the expected product sizes were 93 bp for the wild-type alleles and 113 bp for the mutant ones.

Results from AS-PCR, were also confirmed in N = 252 mosquito samples using newly developed TaqMan genotyping assays for kdr mutations V101G and V1016I (3-plex assay), F1534C (2-plex assay) and S989P (2-plex assay) (S3 Table). Mosquito samples included 192 individuals randomly selected from the resistant populations of Fatick, Touba, Dakar, Louga, Mbour, Matam, Barkédji and Ziguinchor areas and 60 individuals from susceptible laboratory strains (S4 Table). The populations of Kédougou were not tested due to the high susceptibility to pyrethroid. Synthetic gBlocks Gene Fragments (Integrated DNA Technologies, Inc., Coralville, Iowa, USA) wild type and mutant dsDNA sequence controls (S5 Table) were also used to analytically optimize assays in terms of specificity and sensitivity by the application of primer and probe matrices to conclude to the final reaction concentrations given in S3 Table. For validation purposes, we additionally used gDNA extracted from the Cayman *Ae*. *aegypti* resistant strain, in which we successfully detected with our TaqMan assays the mutations F1534C and V1016I which have been previously reported to be present in this strain [31]. Reactions were performed in the Viia7 Real-Time PCR system (Applied Biosystems) using a one-step RT-PCR master mix supplied by FTD (Fast-track diagnostics, Luxembourg) in a total reaction volume of 10 μl. The thermal cycle parameters were: 50°C for 15 min, 95°C for 3 min, and 40 cycles of 95°C for 3 sec and 60°C for 30 sec. Samples were amplified in duplicates and each run always included a non-template control and six control samples produced with gBlocks Gene Fragments wild type and mutant dsDNA sequence controls (Integrated DNA Technologies, Inc., Coralville, Iowa, USA) to include all possible combinations: wild type, heterozygote, mutant, and double mutant samples (for V1016G/I) (S5 Table).

The absence of *kdr* mutations was additionally verified with Sanger sequencing in 80 randomly selected samples all resistant to pyrethroids (10 from each population) and in 10 samples from susceptible control strains. PCRs were carried out in 30 μl reactions containing 1.0 unit of Kapa Taq DNA polymerase (Kapa Biosystems), 0.2 mM dNTPs, 1.5 mM MgCl_2_ and 0.5 μM each of the forward and reverse primers. The amplification consists of an initial heat activation step at 95 ^o^C for 2 min, followed by 35 cycles of 95°C for 30 s, 63°C for 30 s and 72°C for 30 s with a final extension step at 72°C for 2 min. The primers used for domain II (mutations: V10106G/I and S989P) were FII: GGTGGAACTTCACCGACTTC and RII: GGACGCAATCTGGCTTGTTA and for domain III (mutation: F1534C) were FIII: GCTGTCGCACGAGATCATT and RIII: GTTGAACCCGATGAACAACA. The PCR fragments were purified using NucleoSpin Gel and PCR Clean-up (Macherey-Nagel). Nucleotide sequences were determined in purified PCR products at the CeMIA sequencing facility (CEMIA, SA., Greece).

### Total RNA extraction and gene expression analysis by multiplex RT-qPCR

Gene expression analysis was performed by comparing 7 field mosquito populations (Mbour, Fatick, Louga, Touba, Dakar, Matam and Barkédji) unexposed to insecticides with 3 susceptible control laboratory strains (New Orleans, Liverpool and Rockefeller). All samples were previously preserved in RNA later. Total RNA was extracted from pooled mosquito specimens (N = 10 per pool) using the MagSi magnetic beads extraction kit (magtivio b.v., Nuth, The Netherlands) as previously described [32]. The quantity and purity of DNA and total RNA were assessed using a Nanodrop 2000c spectrophotometer (Thermo Scientific). The quality of RNA was assessed by 1.0% w/v agarose gel electrophoresis (S1 Fig).

A total of four 3-plex *Ae*. *aegypti* Detox assays (Detox (A)–Detox (D)) were designed by making use of a three color (FAM-green, HEX-yellow, Atto647N-red) TaqMan probe chemistry. Along with 60S ribosomal protein L8 (*RPL8*) (AAEL000987), *CYP6BB2* (AAEL014893) and *CYP9J26* (AAEL014609) compiled Detox (A), *GSTD4* (AAEL001054) and *CCEae3a* (AAEL023844) Detox (B), *CYP9J28* (AAEL014617) and *CYP9M6* (AAEL001312) Detox (C), and *CYP9J32* (AAEL008846) Detox (D). The *RPL8* gene was included in all four Detox assays, thus allowing robust normalization of each reaction independent of sample volume and other possible reaction variations. Primers and probes for the multiplex TaqMan qPCR assays were designed *de novo* (S3 Table) taking into account the standard guidelines for qPCR assays enhanced with the following criteria: i) Primer(s) in exon junction to avoid DNA amplification and ii) applicability for multiplexing. For the latter criterion, testing of potential primer dimers was performed *via* the Multiple Primer Analyzer software (Thermo Scientific). All oligos were optimized using primer and probe matrices. The analytical parameters of the RT-qPCR reactions (efficiency, linearity, dynamic range, sensitivity) are presented in details in S3 Fig. The %CV of measurements ranged from 0.69%– 6.77%. For validation purposes, we used total RNA extracted from the Cayman resistant laboratory strain, in which a >2.0-folds statistically significant (P < 0.05) overexpression of *CYP6BB2*, *CYP9J28*, *CYP9M6* and *CYP9J32* genes was successfully detected, in line with previously published findings [31,33]. Reactions (10 μL) were carried out using the one-step reverse transcription-qPCR mastermix supplied by FTD (Fast-track diagnostics, Luxembourg) in 96-well plates in the ViiA 7 Real-Time PCR System (Applied Biosystems, Waltham, Massachusets, USA). The exact primer and probe concentrations are provided in Supplementary S6 Table. The thermal cycling protocol was the following: 50°C for 15 min, 95°C for 3 min, and 40 cycles of 95°C for 3 sec and 60°C for 30 sec.

### Statistical analysis

Mosquito mortality was evaluated 24h post insecticide exposure and the susceptibility status was determined according to WHO criteria [26]. Mortality rates ≥ 98% indicated susceptibility, mortality rates between 90% and 97% indicated suspected resistance or tolerance and mortality rates less than 90% indicated resistance to the tested insecticide. When mortality in the control was between 5% and 20%, the mortality rates in test samples were corrected using the Abbott formula [34]. The test result was omitted if the mortality in control was more than 20%. For pyrethroids and DDT, the knock-down times for 50% (KdT 50) and 95% (KdT 95) of tested mosquitoes were calculated using log-probit model with R-Studio software [35]. Calculation of fold-changes, 95% CIs and statistical significance was performed according to the Pfaffl method [36]. Graphs were plotted with the SigmaPlot software (v12.0).

## Results

### Insecticide susceptibility bioassays

#### Mortality rates

**Fig 2:**
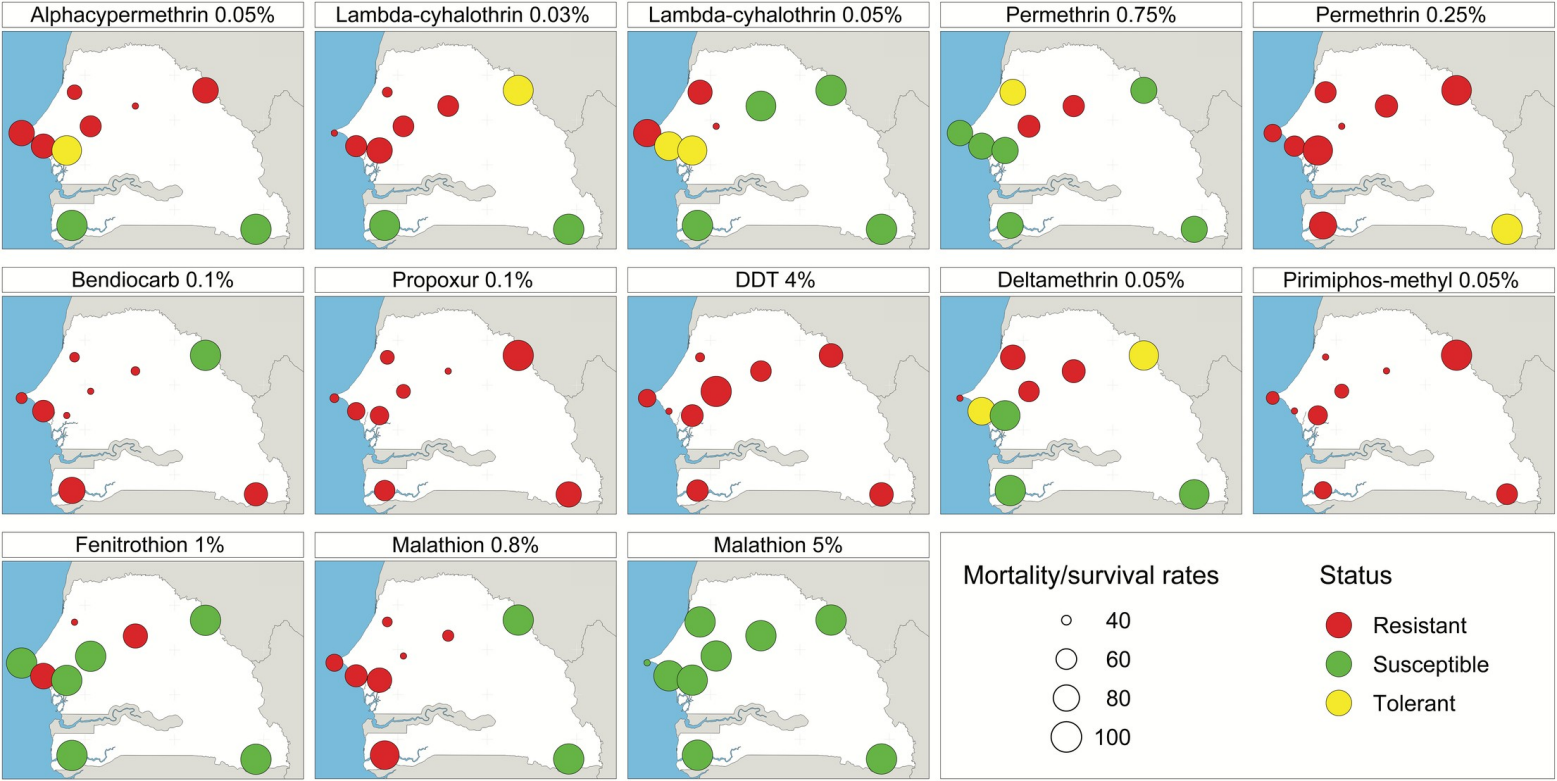
Map showing the resistance status of *Ae*. *aegypti* populations, collected from nine localities of Senegal in 2017–2019, to the four classes of insecticides. This map was built using a shapefile from the free domain of the Geographic Information System (http://www.diva-gis.org) with the R software version 3.3.1 and the package map tools.

Results of insecticide susceptibility tests in *Ae*. *aegypti* populations collected from nine localities of Senegal are presented in Fig 2 and S2 Table.

Susceptibility to 0.75% permethrin was observed in all populations except in those from Barkédji, Louga and Touba which were resistant (respective mortality rates: 61.00%, 96.29% and 66.32%, respectively). All populations were also resistant to 0.25% permethrin (mortality rates < 88.66%), except for the Kédougou population that exhibited a suspected resistance (mortality = 93.3%).

Only the populations from Kédougou and Zinguinchor were susceptible to all type II pyrethroids (deltamethrin, alpha-cypermethrin and lambda-cyhalothrin). Populations from Fatick were susceptible to deltamethrin and those from Barkédji and Matam were susceptible to 0.05% lambda-cyhalothrin. All other populations showed a suspected or confirmed resistance.

Resistance to DDT was detected in all nine populations tested with 24h post exposure. The highest mortality rate was noted in Touba (65.11%) and the lowest was recorded in Louga (10.41%).

All the *Ae*. *aegypti* populations tested were susceptible to 5% malathion, while only those from Kédougou and Matam were susceptible to 0.8% malathion (mortality rates of susceptible populations up to 100%). In contrast, all populations were resistant to 0.05% pirimiphos-methyl (mortality rates less than 37.62%) whereas those from Louga, Mbour and Barkédji strains exhibited resistance to 1% fenitrothion with mortality rates of 70.4%, 89.6% and 87% respectively.

Apart from the Matam populations, that exhibited 100% of mortality with 0.1% bendiocarb, all other populations were resistant to carbamates with mortality rates below 69.3% for 0.1% bendiocarb and 78.18% for 0.1% propoxur.

#### Knock down effects

The knockdown times for 50% and 95% (KdT50 and KdT95) of tested mosquitoes were determined for pyrethroids and organochlorines (Table 1). For each of the insecticides tested, Kédougou and Ziguinchor mosquitoes were the most susceptible and displayed the lowest KdT50 and KdT95 values. The KdT50 and KdT95 values of the resistant populations were considerably higher compared to susceptible populations. The KdT50 and KdT95 values of the populations resistant to 0.05% deltamethrin (Louga, Barkédji, Touba and Dakar), 0.05% alpha-cypermethrin (Louga and Barkédji), 0.75% permethrin (Barkédji), 0.03% lambda-cyhalothrin (Louga, Dakar and Touba) 0.05% lambda-cyhalothrin (Barkédji, Louga, Dakar, Fatick and Touba) were respectively 2.0–7.0 and 3.0–11.0 times higher than that of the Kédougou populations. For 0.25% permethrin and 4% DDT, the KdT50 and KdT95 of all the mosquito populations were higher compared to the other insecticides tested.

**Table 1:** Knockdown time (KDT50 and KDT95) of *Ae*. *aegypti* populations during the 1hour exposure to pyrethroids and organochlorines.

Locality		0.05%	0.05%	0.25%	0.75%	0.03%	0.05%	4% DDT
		delta	alpha	perm	perm	Lambda	Lambda	
Louga	KdT50	31.7	85.7	64.1	26.15	85.72	45.2	-
		[27.2–36.9]	[82.8–88.6]	[61.7–66.7]	[24.3–28]	[82.8–88.6]	[38.6–52.8]	-
	KdT95	53.9	158.6	115.4	46.5	158.6	87.1	-
		[42.3–68.8]	[146.2–172.1]	[104.7–127.2]	[41.6–52]	[146.2–172.1]	[62.9–120.5]	-
Dakar	KdT50	29	40.2	91.9	20.5	90.3	50.5	112.8
		[25.1–33.5]	[39.1–41.3]	[86.9–97.1]	[20.2–20.8]	[84.2–96.8]	[44.9–56.8]	[100.3–126.9]
	KdT95	61.3	96.8	162.2	30.8	163.8	112.6	226.2
		[48.1–78]	[91.1–102.7]	[142.7–184.2]	[29.9–31.8]	[139–192.9]	[85–149]	[176–290.7]
Barkédji	KdT50	23.8	67.8	85.5	44.5	39.4	31.6	112.9
		[20.8–27.1]	[63.2–72.8]	[79–92.5]	[41.8–47.5]	[38.4–40.5]	[31.1–32.2]	[93–136.9]
	KdT95	48.7	152	195.8	132.4	64.6	42	272.5
		[39.4–60.5]	[126.2–183.1]	[160.9–238.2]	[112.8–155.4]	[61.6–67.8]	[40.8–43.2]	[177.4–418.6]
Mbour	KdT50	6.7	30.4	76.8	13.5	54.9	35.8	112.2
		[5.2–8.8]	[25.5–36.2]	[71.5–82.5]	[10.2–17.8)]	[52–58]	[31.8–40.3]	[104.6–120.5]
	KdT95	29.9	68.5	134.7	24.1	120.8	71	181
		[25.1–35.6]	[50.4–3.2]	[112.8–161]	[14.8–38.9]	[105.5–138.4]	[57.6–87.6]	[156.8–209]
Ziguinchor	KdT50	13.6	14.8	53.6	14.1	33	15.7	82.1
		[12.9–14.4]	[14.4–15.3)]	[50.7–56.7]	[13.7–14.4]	[28.8–37.8]	[15.6–15.9]	[82–82.1]
	KdT95	17.5	27.1	81.1	22.8	33	26.2	89.1
		[16.2–19.0]	[25.7–28.5]	[72.2–91]	[21.8–23.9]	[28.8–37.8]	[25.8–26.7]	[88.9–89.3]
Kédougou	KdT50	7.6	11.8	36.3	11.7	16.9	16.6	81.1
		[7–8.3]	[11.6–12.1]	[32.9–39.9]	[11.4–11.9]	[16.2–17.6]	[16.2–16.9]	[81–81.1]
	KdT95	18.3	21.1	67	18.6	27.1	28.7	87.7
		[17.3–19.4]	[20.4–21.9]	[56.7–79.2]	[18.1–19.2]	[25–29.2]	[27.5–29.9]	[87.6–87.9]
Fatick	KdT50	21.7	22.8	39.4	15.3	41.6	22.9	98.1
		[18.3–25.9]	[20.2–25.7]	[36.6–42.3]	[15.3–15.3]	[40.2–43.1]	[21.6–24.2]	[94.3–102]
	KdT95	35.9	36.4	74.6	20.6	73.1	39.4	147.1
		[26.7–48.2]	[29.8–44.5]	[65.3–85.1]	[20.6–20.7]	[68.5–78]	[35.9–43.2]	[134.8–160.5]
Touba	KdT50	35.5	49.5	94.5	34.7	68.7	41.7	65.4
		[33–38.1]	[45.6–53.8]	[94–95]	[31.6–38.1]	[57.7–81.7]	[39.6–43.8]	[57.1–74.9]
	KdT95	79.9	234.4	106.4	64.8	285.3	57.1	150.7
		[69.6–91.8]	[183.6–299.3]	[105.4–107.3]	[55.3–76]	[176.5–461.3]	[52.5–62.2]	[105.5–215.5]
Matam	KdT50	16.5	18.1	36.2	15.1	34.4	21.4	71.5
		[15.9–17.2]	[11.5–28.6]	[33.4–39.3]	[14.4–15.9]	[33.4–35.5]	[20.5–22.4]	[66–77.5]
	KdT95	29.3	52.9	80.4	24	49.2	33.2	160.5
		[27.4–31.3]	[25.6–109.2]	[68.9–93.8]	[22–26.2]	[46.8–51.6]	[30.8–35.8]	[30.3–197.8]

lambda: Lambda-cyhalothrin, alpha: alpha-cypermethrin, delta: deltamethrin, perm: permethrin, KdT: Knock down time in minute, []: confidence interval

### Molecular analysis of resistance mechanisms

#### Assessing the presence and frequency of known target site resistance mutations

A total of 1778 mosquitoes were randomly selected from insecticide resistant and susceptible populations from the nine localities and tested by AS-PCR for the V1016G and F1534C. For each insecticide tested, all mosquitoes or a maximum of 24 individuals were used for genotyping respectively when the total number was less or highest than 24. For both loci, no mutations. No resistant allele was detected in any of the samples. The absence of resistant alleles was also confirmed in N = 252 mosquito samples using newly developed TaqMan genotyping assays (kdr V101G, V1016I, F1534C and S989P). The absence of kdr mutations was additionally verified in N = 80 randomly selected samples all resistant to pyrethroids (10 from each population) and in 10 New Orleans *Ae*. *aegypti* susceptible strain as a control) with Sanger sequencing (S2 Fig). Thus, all samples tested were found to be wild type for all the target site resistance loci previously implicated in pyrethroid resistance.

#### Determination of expression levels of detoxification genes previously implicated in metabolic resistance

**Fig 3:**
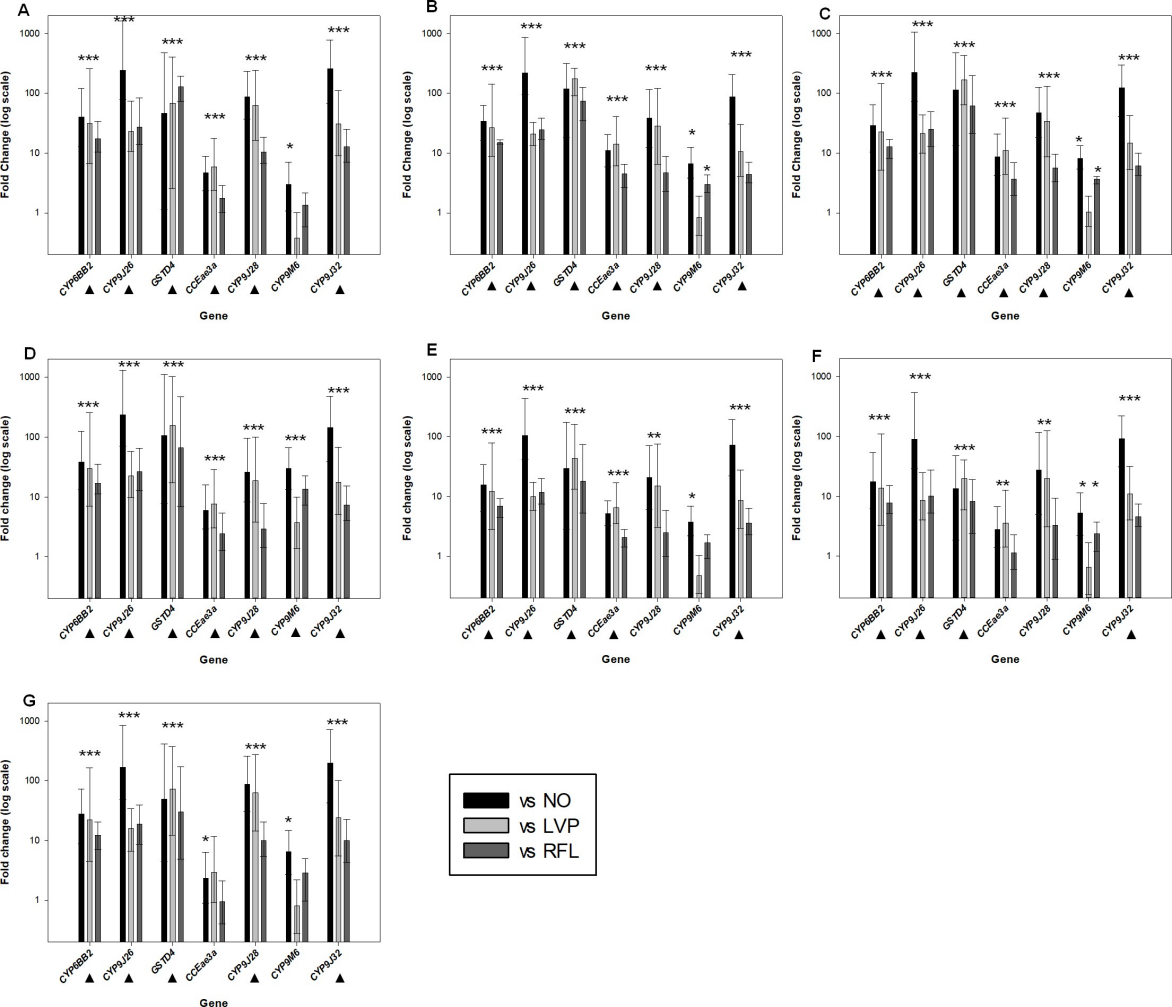
Expression analysis of detoxification genes in the seven resistant mosquito populations (respectively Mbour, Fatick, Louga, Touba, Dakar, Matam, and Barkédji). Error bars indicate 95% CIs. Stars denote statistically significant upregulation.

The expression levels of seven major detoxification genes (*CYP6BB2*, *CYP9J26*, *CYP9J28*, *CYP9J32*, *CYP9M6*, *CCEae3a* and *GSTD4*) implicated in insecticide resistance in *Ae*. *aegypti*, were assessed with newly developed multiplex TaqMan RT-qPCR assays in seven localities of the study and compared with three *Ae*. *aegypti* insecticide susceptible strains (Fig 3 and S7 Table).

Three cytochrome P450s genes (*CYP6BB2*, *CYP9J26* and *CYP9J32*) were found to be significantly overexpressed in all seven tested localities. More precisely, *CYP6BB2* was upregulated in Mbour (17.5–39.8 folds), Fatick (15.1–34.3 folds), Louga (12.8–29.0 folds), Touba (16.9–38.3 folds), Dakar (6.88–15.63 folds), Matam (7.71–17.5 folds) and Barkédji (12.3–28.0 folds). *CYP9J26* was overexpressed in Mbour (23.3–244 folds), Fatick (20.9–220 folds), Louga (21.3–224 folds), Touba (22.3–235 folds), Dakar (9.90–104 folds), Matam (8.64–90.8 folds in) and Barkedji (16.1–169 folds). *CYP9J32* was upregulated in Mbour (12.7–257 folds), Fatick (4.37–88.0 folds), Louga (6.08–123 folds), Touba (7.22–145 folds), Dakar (3.58–72.1 folds), Matam (4.53–91.3 folds) and Barkédji (9.93–200 folds). The cytochrome P450 gene *CYP9J28* showed a significant overexpression in five out of seven localities (Mbour: 10.5–86.8 folds, Fatick: 4.70–38.9 folds, Louga: 5.68–47.0 folds, Touba: 2.88–25.8 folds and Barkédji: 10.1–86.9 folds), whereas the *CYP9M6* gene was upregulated only in one locality (Touba: 3.72–29.8 folds).

The esterase *CCEae3a* gene was upregulated in five out of seven populations, i.e. Mbour (1.76–4.67 folds), Fatick (4.54–14.2 folds), Louga (3.65–11.1 folds), Touba (2.41–7.52 folds) and Dakar (2.09–6.52 folds).

The *GSTD4* gluatthione-S-transferase gene showed a pattern of consistent upregulation across all seven localities of the study. It was overexpressed in Mbour (46.5–129 folds), Fatick (73.6–177 folds), Louga (62.0–115 folds), Touba (65.6–157 folds), Dakar (18.2–43.8 folds), Matam (8.28–19.9 folds), and Barkédji (30.3–49.0 folds).

## Discussion

Although *Ae*. *aegypti* is the main epidemic vector of several arboviral diseases in urban areas of Senegal, little is known about its susceptibility to currently used insecticides.

In the present study, all *Ae*. *aegypti* populations tested were resistant to DDT in concordance with previous studies performed in Senegal [15]. Resistance to DDT could be the result of a direct impact of previous vector control programs adopted widely applied within the country [37]. Our results showed that all *Ae*. *aegypti* populations investigated were susceptible to 0.75% permethrin except those from Barkédji, Louga, and Touba. Other studies on *Ae*. *aegypti* populations from urban areas of Senegal and Nigeria showed similar susceptibility to 0.75% permethrin [15,38,39]. In contrast, resistant populations have been reported in Asia [40].

Our results show also that only the *Ae*. *aegypti* populations from Kédougou and Ziguinchor were susceptible to type II pyrethroids (deltamethrin, alpha-cypermethrin and lambda-cyhalothrin). This finding could be explained by the predominance in the southern area of Senegal of *Ae*. *ae*. *formosus*, the normally forest-dwelling subpopulation of *Ae*. *aegypti*, which uses tree holes for oviposition and larval development sites in the forest, but were recently also found in the domestic environment of this area. This recent occurrence in the domestic environment, where discarded containers act as breeding sites, is believed to be an adaptation to deforestation for agricultural, gold mining and other human activities. The domestication of wild *Ae*. *aegypti* populations over southern Senegal is also probably a response to a urbanization process of the region along with a growing human population and an increased presence of discarded containers from growing consummation of modern manufactured and industrial foods and goods [41]. This morphotype is known as less domestic and exophilic [42,43]. It is also known as primarily zoophilic [42] while a recent study exhibited an antrhopophilic tendancy [43]. This population was probably less exposed to insecticides than the highly domesticated and anthropophilic population of *Ae*. *ae*. *aegypti* predominant in the other localities investigated. Another explanation could be a weak capacity for resistance selection of the form *Ae*. *aegypti formosus* prevailing in this southern region [44], known as a cotton growing area with intense use of insecticides.

In addition, malaria control strategies using insecticides impregnated nets and indoor residual spraying could also explain the high resistance of *Ae*. *aegypti* populations to pyrethroids in the center of Senegal and their susceptibility in the Northern regions. Indeed, these malaria control tools are still intensely used in the center (where malaria is still a public health problem) and less applied in the north where malaria has almost eradicated [45]. Due to the exophilic behavior of the malaria vectors in southern Senegal, IRS was not applied in this area up to 2019 and only bed nets were used.

With the wide spread of mosquito pyrethroid resistance globally [46,47], it is important to carry out additional monitoring studies, since it is the most common class of insecticide for vector control [48].

Similarly to pyrethroids, the mosquitoes tested in this study were highly resistant to the organophosphates used, except for 5% malathion and 1% fenitrothion. Analogous results were reported during previous studies with the same insecticides and diagnostic doses in Burkina Faso [21], and also with 1% fenitrothion in Cape Verde and Senegal [15] and with 0.5% fenitrothion in Cameroon [49]. The high dose of 5% malathion used (6 times higher than the diagnostic dose for *Aedes* resistance) may explain the susceptibility for all populations. In addition, these two insecticides (1% fenitrothion and 5% malathion) belong to an insecticide class no longer in use for vector control in Senegal. Therefore, these two insecticides can still be used to control *Ae*. *aegypti* populations, at least in some localities of Senegal.

All populations tested were resistant to propoxur which is the main product used for interventions during reactive campaigns in response to epidemics [50]. This documented resistance is the result of long-term use of this product. Dia et al. [15] have shown the incipient resistance status of *Ae*. *aegypti* populations from Dakar during the 2009 dengue outbreak. Our findings are consistent with previous results obtained in Côte d’Ivoire, Cameroon and Burkina Faso [21,49,51], although susceptibility to this insecticide is still reported to date in *Ae*. *aegypti* in some localities of Congo [52].

Despite the resistance observed to pyrethroids, the remarkable absence of any of the tested and most known target site pyrethroid resistance mutations (F1534C, V1016I, V1016G and S989P) is reported. This is in contrast to previous studies in other geographical regions in Africa, where two of the mutations assayed here (F1534C and V1016I) were detected previously in Ghana and Burkina Faso, and more recently in Angola and Cape Verde [21,23,53]. The mutation V410L, recently detected for the first time in Africa in Angola by Ayres et al. [22] was not investigated in this study. Further *kdr* surveys should include this mutation to better characterize the *kdr* distribution in Africa. However, similar results to our observations in Senegal were found in Cameroon, Congo [52] and Central African Republic [54] where kdr mutations were absent in *Ae*. *aegypti* populations resistant to pyrethroids [52]. The possible explanations for these results are: 1) the known *kdr* mutations are not involved in the observed resistance in *Ae*. *aegypti* populations tested in Senegal and Cameroon, 2) other mechanisms like metabolic resistance are probably in place.

Several studies showed that detoxification proteins like cytochrome P450s, are associated with pyrethroid resistance in *Ae*. *aegypti* [13,55,56]. However, until recently, the geographic distribution of this metabolic resistance was limited to Asia and America, as it was regularly reported in several countries like Thailand, Mexico, Cuba, Puerto Rico, Peru, Singapore and Vietnam [57]. In Africa, while suspected by several authors, based mainly on results from bioassays using synergists, few molecular data were available in *Ae*. *aegypti*. Our data clearly show high levels of *CYP9J26*, *CYP9J32* and *CYP6BB2* expression consistently found across resistant populations of *Ae*. *aegypti*, while *CYP9J28* is significantly overexpressed in five out of seven populations. *CYP9J32* and *CYP9J26* have been confirmed to metabolize both Type I and Type II pyrethroids and *CYP6BB2* to metabolize Type I pyrethroids *in vitro* [57]. Thus, our data strongly indicate that CYP-mediated detoxification is the main mechanism of pyrethroid resistance currently operating in Senegal. The glutathione transferase *GSTD4* gene was also significantly overexpressed in all of the *Ae*. *aegypti* populations investigated; association of *GSTD4* overexpression and pyrethroid resistance has been documented in resistant *Ae*. *aegypti* populations from Singapore [58,59]. The esterase *CCEae3a* gene was found overexpressed in resistant *Ae*. *aegypti* in five localities (Louga, Touba, Fatick, Dakar and Mbour) and could possibly be linked with the observed organophosphate resistance. It is known that CCEs catalyze the hydrolysis of ester bonds and are associated with organophosphate and carbamate resistance, either through sequestration or direct metabolism [12].

## Conclusion

Our study shows that *Ae*. *aegypti* populations from Senegal are resistant to insecticides from four insecticide classes (pyrethroids, organochlorines, carbamates and organophosphates). This constitutes an alarming phenomenon, since these insecticides remain the only ones used to confront disease vectors, especially in low-and middle-income countries like Senegal. However, this requires further consideration, since many diagnostic doses used are not specific for *Aedes* mosquitoes. The observed resistance was exclusively due to metabolic resistance in light of the absence of the known and well-studied *kdr* mutations F1534C, V1016G, V1016I, S989P and the overexpression of several detoxification genes. It is important to estimate the intensity of resistance but also to investigate the possibility of testing newly proposed concentrations, emerging insecticides (such as clophenapyr and clothianidrin) and other alternative methods (such as Bacillus *thuringiensis var*. *israelensis* (Bti), *Wolbachia* infection, sterile insect techniques and genetic manipulation) in African countries where epidemics can have devastating effects.

## Supporting information

S1 Fig Agarose gel electrophoresis for total RNA extracted from the study’s mosquito samples. The presence of distinct ribosomal bands and the absence of degradation products shows that total RNA was intact, suitable for downstream analyses.
(TIFF)

S2 Fig Representative electropherograms of *Ae*. *aegypti* samples genotyped for kdr mutations. All samples were wild type for all the kdr mutations that were screened for (V1016G/I- domain II, S989P-domain II, F1534C- domain III)
(TIFF)

S3 Fig Analytical properties of the novel multiplex RT-qPCR assays (Detox A-D) for gene expression analysis. Standard curves performed in multiplex (left panel) versus singleplex (right panel) format.
(TIF)

S1 Table Geographical location and population size of study sites. (DOCX)

S2 Table Total numbers and mortality rates of *Aedes aegypti* after exposure to four class insecticides in 2017–2019 in nine localities in Senegal using WHO standard bioassay. (DOCX)

S3 Table Primers and probes used for the TaqMan kdr genotyping. F: Forward; R: Reverse; P: Probe; wt: wild type; mt: mutant
(DOCX)

S4 Table Incidence of resistances alleles in different populations of *Ae*. *aegypti* mosquitoes assayed by TagMan qPCR. (DOCX)

S5 Table Synthetic Blocks Gene Fragments used as controls for the newly developed Taqman kdr assays. Notes: 1. For universal wild-type FF, SS, VV control use GB1. 2. For mutant CC, PP, II control use GB2 3. For mutant GG control use GB3. 4. For heterozygous FC, SP, VI control mix equal amounts of GB1 and GB2. 5. For heterozygous VG control mix equal amounts of GB1 and GB3. 6. For double mutant VI control mix equal amounts of GB2 and GB3.
(DOCX)

S6 Table Primers and TaqMan probes of the novel multiplex RT-qPCR assays for gene expression analysis. F: Forward, R: Revers, P: Promotor
(DOCX)

S7 Table Expression analysis of the detoxification genes analyzed in the seven resistant mosquito populations compared to three susceptible mosquito strains (New Orleans, Liverpool, and Rockefeller). Bold letters indicate statistical significance for all three comparisons. NO: New Orleans susceptible lab strain; LV: Liverpool black–eyed susceptible lab strain; RF: Rockefeller susceptible lab strain; CI: confidence interval.
(DOCX)
