# Supplementary figures and images for: Insecticide resistance status and mechanisms in Aedes aegypti populations from Senegal
Source: PLoS Negl Trop Dis. 2021 May 10;15(5):e0009393. doi: 10.1371/journal.pntd.0009393 (PMC8136859; doi:10.1371/journal.pntd.0009393)

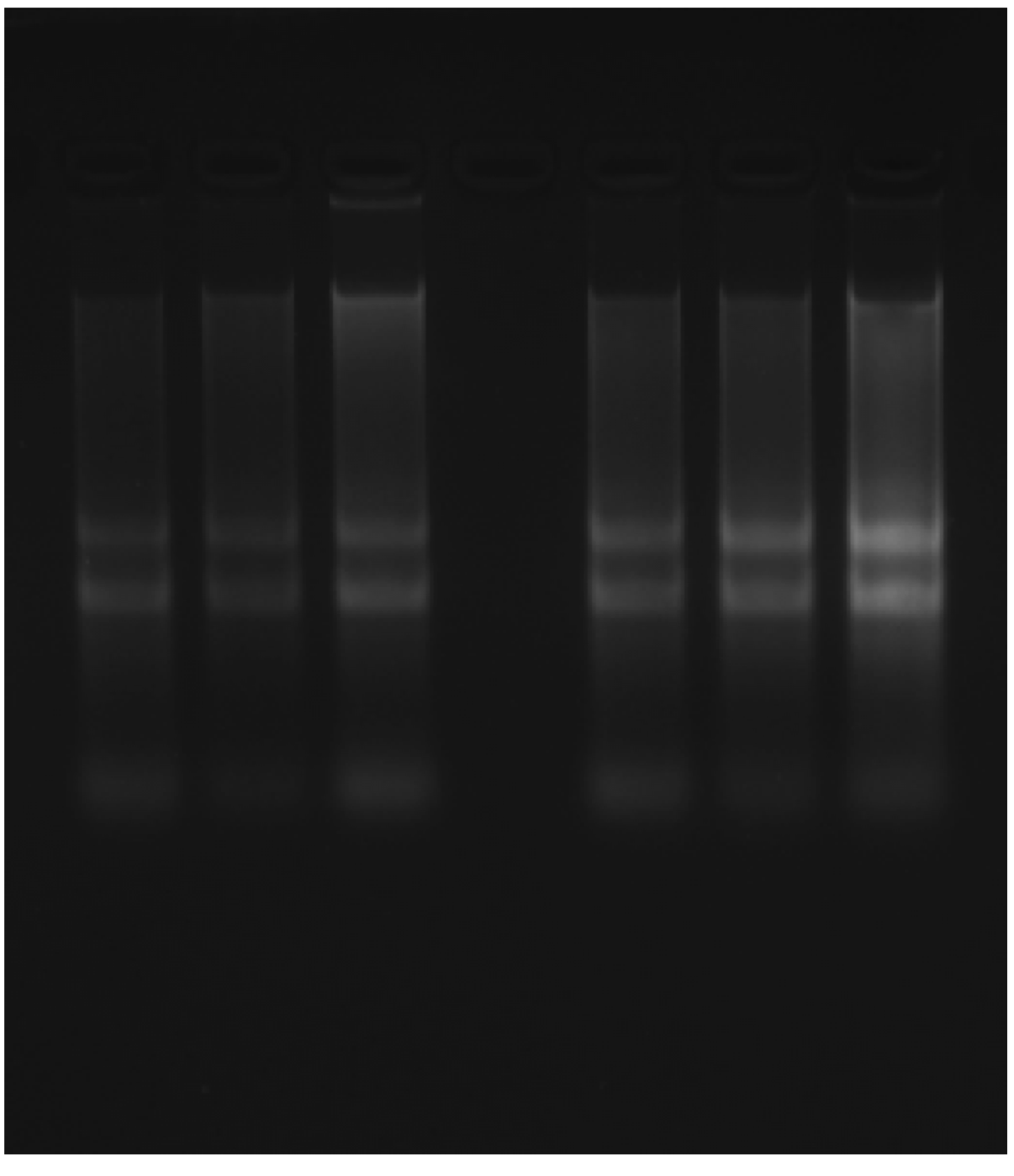

Supplement: S1 Fig — The presence of distinct ribosomal bands and the absence of degradation products shows that total RNA was intact, suitable for downstream analyses. (TIFF) [file pntd.0009393.s001.tiff]

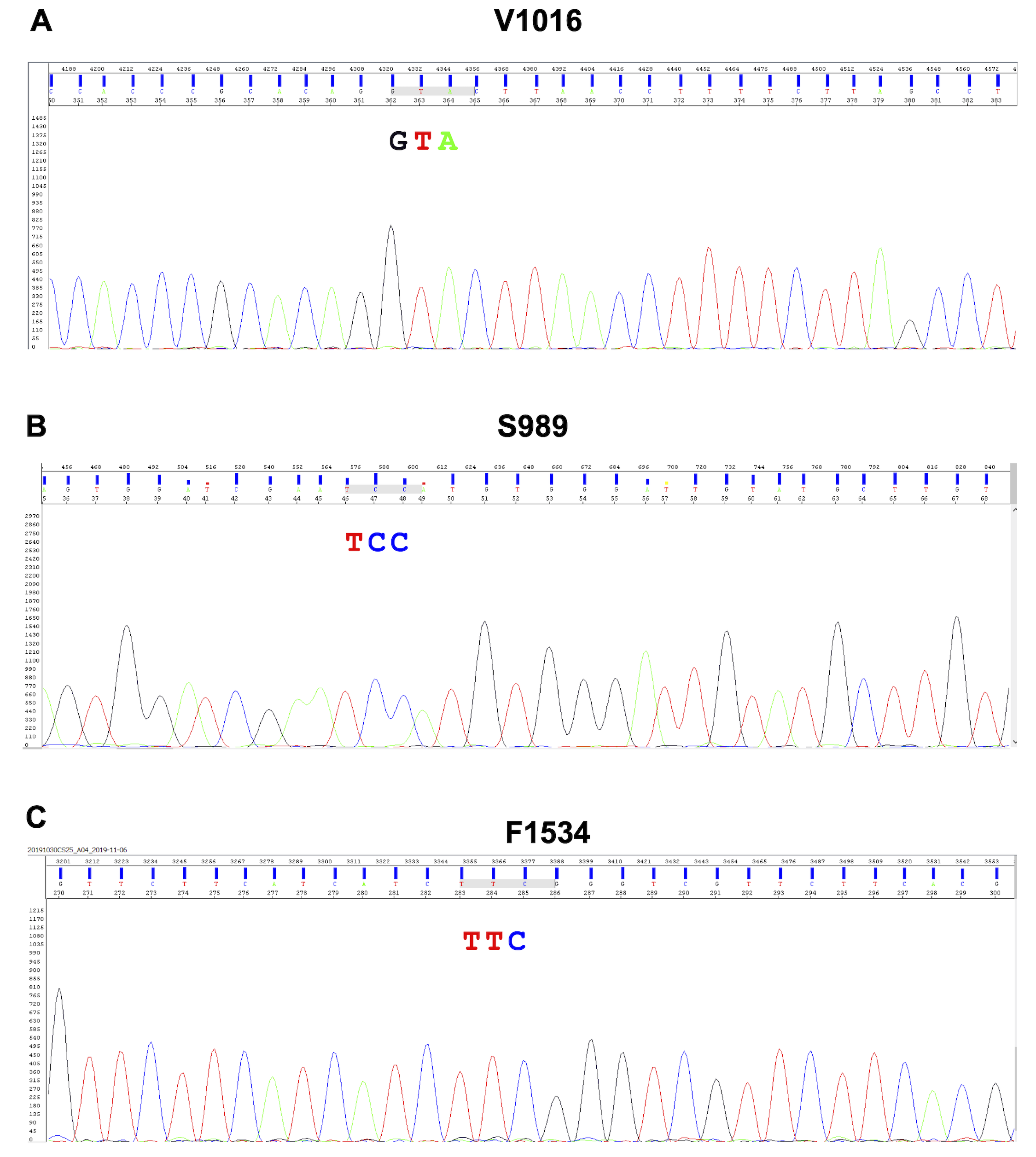

Supplement: S2 Fig — All samples were wild type for all the kdr mutations that were screened for (V1016G/I- domain II, S989P-domain II, F1534C- domain III) (TIFF) [file pntd.0009393.s002.tiff]

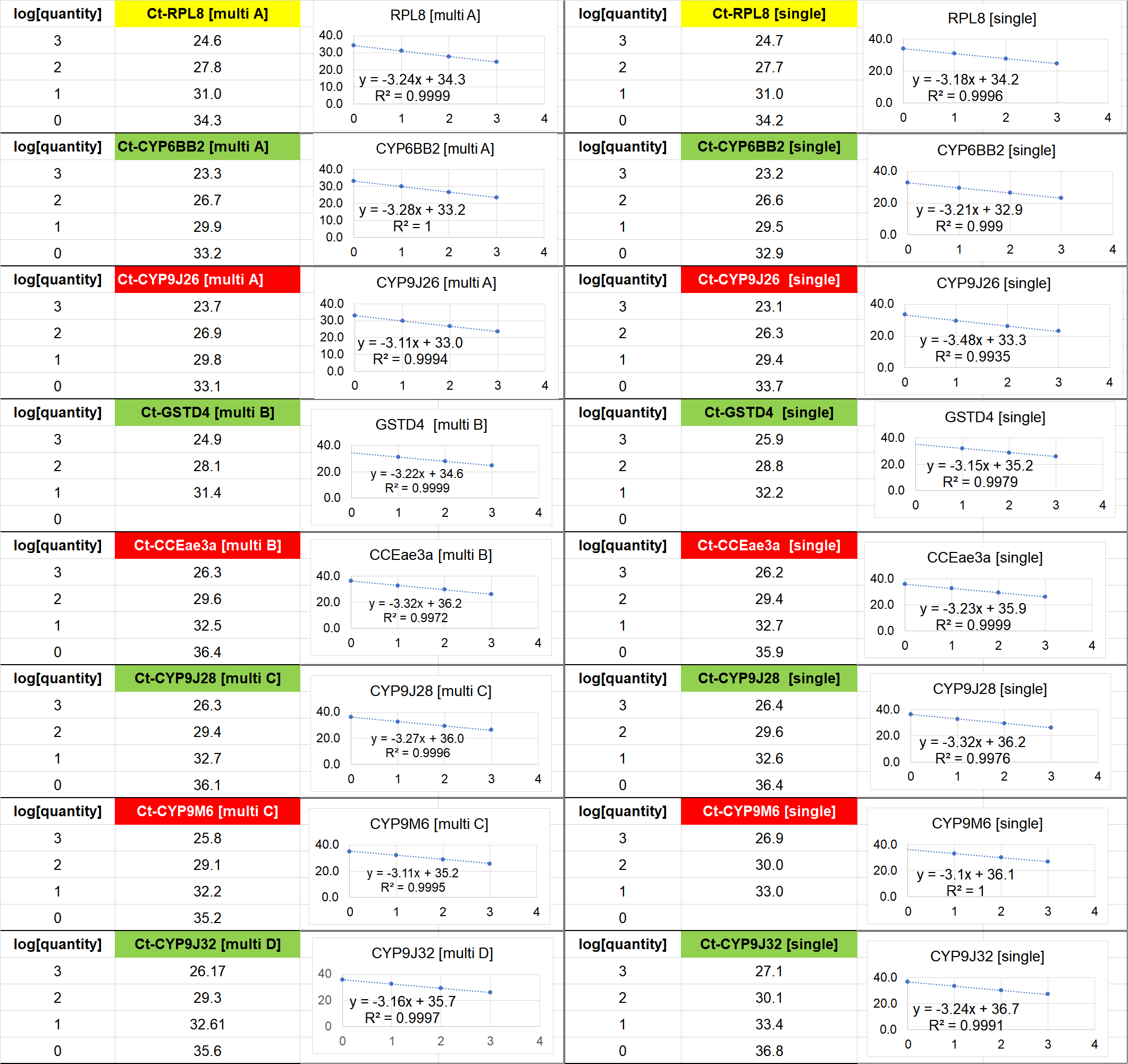

Supplement: S3 Fig — Analytical properties of the novel multiplex RT-qPCR assays (Detox A-D) for gene expression analysis. Standard curves performed in multiplex (left panel) versus singleplex (right panel) format. (TIF) [file pntd.0009393.s003.tif]
