# Supplementary material for: Insecticide resistance status and mechanisms in Aedes aegypti populations from Senegal
Source: PLoS Negl Trop Dis. 2021 May 10;15(5):e0009393. doi: 10.1371/journal.pntd.0009393 (PMC8136859; doi:10.1371/journal.pntd.0009393)
Supplement: S1 Table — (DOCX) [file pntd.0009393.s004.docx]

**S1 table Geographic and population size of study sites**

| **Position** | **Phytogeographic** | **Study site** | **Population 2016** | **Geographic coordinats** |
| --- | --- | --- | --- | --- |
| Center | Sahelo-Sudanian | [Dakar](https://fr.wikipedia.org/wiki/Dakar) | 1 252 786 | 14°40' 22,5"N, 17°26'37"W |
|  |  | Touba | 93 4428 | 14°51'00" N, 15°53'00" W |
|  |  | [Mbour](https://fr.wikipedia.org/wiki/Mbour) | 252 645 | 14°25'7,6" N, 16°57'23,1" W |
|  |  | [Louga](https://fr.wikipedia.org/wiki/Louga) | 113 410 | 15°37'16,5" N, 16°13'67,6"W |
|  |  | [Barkedji](https://fr.wikipedia.org/wiki/Dahra_(S%C3%A9n%C3%A9gal)) | 13 589 | 15°16'37,4" N, 15°51'46,8"W |
|  |  | [Fatick](https://fr.wikipedia.org/wiki/Fatick) | 48 459 | 14°36'67" N 16°13'33" W |
| North | Sahelian | [Matam](https://fr.wikipedia.org/wiki/Matam_(S%C3%A9n%C3%A9gal)) | 19 304 | 15°39'47,4" N, 13°15'18,7"W |
| South | Sudano-Guinean | [Ziguinchor](https://fr.wikipedia.org/wiki/Ziguinchor) | 225 024 | 12°33'47,4 N, 16°17'34,6"W |
|  |  | [Kédougou](https://fr.wikipedia.org/wiki/K%C3%A9dougou) | 18 860 | 12°33'45,3" N, 12°10'31,9"W |
