# Supplementary material for: Insecticide resistance status and mechanisms in Aedes aegypti populations from Senegal
Source: PLoS Negl Trop Dis. 2021 May 10;15(5):e0009393. doi: 10.1371/journal.pntd.0009393 (PMC8136859; doi:10.1371/journal.pntd.0009393)
Supplement: S2 Table — (DOCX) [file pntd.0009393.s005.docx]

| Locality | 0.05% deltame  thrine | 0.05% alpha  cypermethrin | 0.25% permehrin | 0.75% permehrin | 0.03% lambdacyhalothrin | 0.05% lambdacyhalothrin | 4% DDT | 1% fenitrothion | 5% malathion | 0,8% malathion | 0.05% primiphos-methyl | 0.1% propoxur | 0.1% bendiocarbe |
| --- | --- | --- | --- | --- | --- | --- | --- | --- | --- | --- | --- | --- | --- |
| Louga | **98 ( 80.61)** | **110 (46.36)** | **113 (38.05)** | **108 (96.29)** | **115 ( 27.73)** | **98 (72.44)** | **96 (10.41)** | **115 (75.73)** | **101 (100)** | **100 (6)** | **112 (0)** | **116 (7.75)** | **103 (1.94)** |
| Dakar | **97 (53.6)** | **105 (78.09)** | **110 (23.63)** | **102 (97.14)** | **110 (20)** | **104 (85.57** | **100 (22)** | **109 (100)** | **99 (98)** | **110 (23.63)** | **105(2.85)** | **110 (0.91)** | **100 (4)** |
| Barkedji | **105 (76.19)** | **100 (39)** | **100 (44)** | **100 (61)** | **106 (49.1)** | **112 (97.23)** | **88 (29.54)** | **100 (87)** | **100 ( 100)** | **100 (8)** | **102 (0)** | **100 (0)** | **100 (1)** |
| Mbour | **100 (90)** | **101 (73.26)** | **100 (35)** | **90 (100)** | **101 (50.49)** | **103 (91.26)** | **93 (9.67)** | **106 (89.62)** | **103 (100)** | **100 (46)** | **100 (0)** | **100 (17)** | **100 (41)** |
| Ziguinchor | **98 (100)** | **100 (100)** | **104 (71.15)** | **100 (100)** | **108 (97.22)** | **100 (100)** | **100 (32)** | **106 (100)** | **109 (100)** | **106 (87.73)** | **100 (8)** | **99 (27.27)** | **101 (69.3)** |
| Kedougou | **104 (97.11)** | **111 (100)** | **105 (93.33)** | **107 (100)** | **101 (99)** | **98 (100)** | **88 (38.63)** | **106 (100)** | **104 (100)** | **105 (100)** | **101 (13.86)** | **108 (49.07)** | **100 (50)** |
| Fatick | **102 (98)** | **98 (95.91)** | **103 (86.4)** | **98 (100)** | **102 (70.58)** | **100 (94)** | **100 (33)** | **100 (100)** | **92 (100)** | **107 (58.87)** | **103 (10.67)** | **100 (20)** | **100 (0)** |
| Touba | **97 (70.10)** | **105 (62.20)** | **101 (3.90)** | **98 (66.32)** | **86 (48.63)** | **93 (48.80)** | **86 (65.11)** | **100 (100)** | **100 (100)** | **100 (4)** | **103 (3.88)** | **102 (7.84)** | **100 (0)** |
| Matam | **103 (96.11)** | **106 (78.3)** | **106 (88.66)** | **110 (100)** | **109 (96.33)** | **90 (100)** | **111 (38.73)** | **100 (100)** | **100 (100)** | **100 (100)** | **101(37.62)** | **110 (78.18)** | **106 (100)** |

Number mosquito tested; Numbers in brackets indicate the mortality rate (%)
