# Supplementary material for: Insecticide resistance status and mechanisms in Aedes aegypti populations from Senegal
Source: PLoS Negl Trop Dis. 2021 May 10;15(5):e0009393. doi: 10.1371/journal.pntd.0009393 (PMC8136859; doi:10.1371/journal.pntd.0009393)
Supplement: S3 Table — F: Forward; R: Reverse; P: Probe; wt: wild type; mt: mutant (DOCX) [file pntd.0009393.s006.docx]

**S3 Table Primers and probes used for the TaqMan kdr genotyping**

| **Assay** | **Oligo Name** | **Sequence** | **Dye labels** | **Optimized**  **Reaction concentration (nM)** |
| --- | --- | --- | --- | --- |
| kdr_F1534C | 1534_F | CGAGACCAACATCTACATGTACCT | none | 400 |
|  | 1534_R | GTCGATGATGACACCGATGA | none | 500 |
|  | F1534_P-wt | AACGACCCGAAGATGA | HEX - MGB | 400 |
|  | 1534C_P-mt | ACGACCCGCAGATGA | FAM - MGB | 400 |
| kdr_S989P | 989_F | CATGATCGTGTTCCGGGTATT | none | 300 |
|  | 989_R | CACGTCACCCACAAGCATACA | none | 600 |
|  | S989_P-wt | ATCGAATCCATGTGGGA | HEX - MGB | 500 |
|  | 989P_Probe-mt | AGTGGATCGAACCCA | FAM - MGB | 50 |
| kdr_V1016G/I | 1016_Forward | ACCGACAAATTGTTTCCCAC | none | 400 |
|  | 1016_Reverse | GGACAAAAGCAAGGCTAAGAA | none | 300 |
|  | V1016_P-wt | CACAGGTACTTAACCT | HEX - MGB | 250 |
|  | 1016G_P-mt1 | CACAGGGACTTAACC | FAM -MGB | 50 |
|  | 1016I_P-mt2 | CGCACAGATACTTAA | CY5 - MGB | 600 |

F: Forward; R: Reverse; P: Probe; wt: wild type; mt: mutant
