# Supplementary material for: Insecticide resistance status and mechanisms in Aedes aegypti populations from Senegal
Source: PLoS Negl Trop Dis. 2021 May 10;15(5):e0009393. doi: 10.1371/journal.pntd.0009393 (PMC8136859; doi:10.1371/journal.pntd.0009393)
Supplement: S4 Table — (DOCX) [file pntd.0009393.s007.docx]

**S4 Table** Incidence of resistance alleles in different populations of *Ae. aegypti* mosquitoes, assayed by TaqMan qPCR.

| **Population (insecticide resistance)** | **Sample size (alleles)** | **Resistant mutation allelic frequencies (hetero/homo)** | | | |
| --- | --- | --- | --- | --- | --- |
|  |  | **Pyrethroids** | | | |
|  |  | **% F1534C** | **%V1016G** | **% V1016I** | **% S989P** |
| New Orleans, Susceptible lab strain | 40 | 0.0 (0/0) | 0.0 (0/0) | 0.0 (0/0) | 0.0 (0/0) |
| Liverpool, Susceptible lab strain | 40 | 0.0 (0/0) | 0.0 (0/0) | 0.0 (0/0) | 0.0 (0/0) |
| Rockefeller susceptible laboratory strain | 40 | 0.0 (0/0) | 0.0 (0/0) | 0.0 (0/0) | 0.0 (0/0) |
| Fatick (permethrin) | 48 | 0.0 (0/0) | 0.0 (0/0) | 0.0 (0/0) | 0.0 (0/0) |
| Louga (deltamethrin) | 48 | 0.0 (0/0) | 0.0 (0/0) | 0.0 (0/0) | 0.0 (0/0) |
| Touba (deltamethrin) | 48 | 0.0 (0/0) | 0.0 (0/0) | 0.0 (0/0) | 0.0 (0/0) |
| Dakar (deltamethrin) | 48 | 0.0 (0/0) | 0.0 (0/0) | 0.0 (0/0) | 0.0 (0/0) |
| Matam (alphacypermethrin) | 48 | 0.0 (0/0) | 0.0 (0/0) | 0.0 (0/0) | 0.0 (0/0) |
| Barkedji (permethrin) | 48 | 0.0 (0/0) | 0.0 (0/0) | 0.0 (0/0) | 0.0 (0/0) |
| Ziguinchor (permethrin) | 48 | 0.0 (0/0) | 0.0 (0/0) | 0.0 (0/0) | 0.0 (0/0) |
| Mbour (pyrethroids) | 48 | 0.0 (0/0) | 0.0 (0/0) | 0.0 (0/0) | 0.0 (0/0) |
