# Supplementary material for: Insecticide resistance status and mechanisms in Aedes aegypti populations from Senegal
Source: PLoS Negl Trop Dis. 2021 May 10;15(5):e0009393. doi: 10.1371/journal.pntd.0009393 (PMC8136859; doi:10.1371/journal.pntd.0009393)
Supplement: S5 Table — Notes: 1. For universal wild-type FF, SS, VV control use GB1. 2. For mutant CC, PP, II control use GB2 3. For mutant GG control use GB3. 4. For heterozygous FC, SP, VI control mix equal amounts of GB1 and GB2. 5. For heterozygous VG control mix equal amounts of GB1 and GB3. 6. For double mutant VI control mix equal amounts of GB2 and GB3. (DOCX) [file pntd.0009393.s008.docx]

**S5 Table Primers and TaqMan probes of the novel multiplex RT-qPCR assays for gene expression analysis**

| **Assay** | **Name** | **Sequence5’-3’** | **Dyes 5’-3’** | **Optimized Reaction concentration (nM)** |
| --- | --- | --- | --- | --- |
| DETOX (A)-(D) [normalizer] | RPL8_F  RPL8_R  RPL8_P | GAAGGGAACCGTCAAGCAAATC  TCACGGAAGTGGACAACCG  ATGATCCAGGTCGTGGTGCCCCG | none  none  HEX-BHQ1 | 200  500  200 |
| DETOX (A) | CYP6BB2_F  CYP6BB2_R  CYP6BB2_P | GGCGAGGGAATCACGATGAA  GTACTTCCGTAGGGTTTCACTGAC  CCGTGAAGAAAATGAAACACTGCGCG | none  none  HEX-BHQ1 | 400  500  200 |
| DETOX (A) | CYP9J26_F  CYP9J26_R  CYP9J26_P | ACAACAAATATCCTGGAGTGAAAGT  CGAGAACAGCGTCTTGCGAA  CGCGATCCGGAGTTGATCAAGC | none  none  ATTO647N-BHQ2 | 400  600  300 |
| DETOX (B) | GSTD4_F  GSTD4_R  GSTD4_P | AGCCGGAATTTTTGAAGATCA  AGATGGCACGCGATTCG  ACGGTTCCCACACTGGCAGTAGGC | none  none  HEX-BHQ1 | 600  600  400 |
| DETOX (B) | CCEae3a _F  CCEae3a _R  CCEae3a_P | TGGATGCAGTTTCCAAAACAC  GTGCACTCATGAGGGTTTCGTA  TAGGCTGTGTAGCAGAGAGCGATGATGAAA | none  none  ATTO647N-BHQ2 | 500  600  500 |
| DETOX (C) | CYP9J28_F  CYP9J28_R  CYP9J28_P | GACAAGTACCGAGGAGTCAAAGTTT  TTAACGGCCACCTGCTTGAT  ACGTACGTCATTCGCGATCCGGA | none  none  HEX-BHQ1 | 500  600  400 |
| DETOX (C) | CYP9M6_F  CYP9M6_R  CYP9M6_P | CGTGATCTGTTTCAAAAGCTTGG  CCAACTGCTTTCCCCTTTTG  TCGGTGCACAATCCAAACAACGAGTT | none  none  ATTO647N-BHQ2 | 400  200  400 |
| DETOX (D) | CYP9J32_F  CYP9J32_R  CYP9J32_P | CTACTTCCACGACAAGCCGATAC  GTCATATCAAACAGTCCAAAAATCTTAGC  TTCCGCTCTTGGGCAGTACCGGTC | none  none  HEX-BHQ1 | 500  600  400 |

F: Forward, R: Reverse, P: Probe
