# Supplementary material for: Insecticide resistance status and mechanisms in Aedes aegypti populations from Senegal
Source: PLoS Negl Trop Dis. 2021 May 10;15(5):e0009393. doi: 10.1371/journal.pntd.0009393 (PMC8136859; doi:10.1371/journal.pntd.0009393)
Supplement: S6 Table — F: Forward, R: Revers, P: Promotor (DOCX) [file pntd.0009393.s009.docx]

**S6 Table Primers and TaqMan probes of the novel multiplex RT-qPCR assays for gene expression analysis**

| **Assay** | **Name** | **Sequence5’-3’** | **Dyes 5’-3’** | **Optimized Reaction concentration (nM)** |
| --- | --- | --- | --- | --- |
| DETOX (A)-(D) [normalizer] | RPL8_F  RPL8_R  RPL8_P | GAAGGGAACCGTCAAGCAAATC  TCACGGAAGTGGACAACCG  ATGATCCAGGTCGTGGTGCCCCG | none  none  HEX-BHQ1 | 200  500  200 |
| DETOX (A) | CYP6BB2_F  CYP6BB2_R  CYP6BB2_P | GGCGAGGGAATCACGATGAA  GTACTTCCGTAGGGTTTCACTGAC  CCGTGAAGAAAATGAAACACTGCGCG | none  none  HEX-BHQ1 | 400  500  200 |
| DETOX (A) | CYP9J26_F  CYP6BB2_R  CYP6BB2_P | ACAACAAATATCCTGGAGTGAAAGT  CGAGAACAGCGTCTTGCGAA  CGCGATCCGGAGTTGATCAAGC | none  none  ATTO647N-BHQ2 | 400  600  300 |
| DETOX (B) | GSTD4_F  GSTD4_R  GSTD4_P | AGCCGGAATTTTTGAAGATCA  AGATGGCACGCGATTCG  ACGGTTCCCACACTGGCAGTAGGC | none  none  HEX-BHQ1 | 600  600  400 |
| DETOX (B) | CCEae3a _F  CCEae3a _R  CCEae3a_P | TGGATGCAGTTTCCAAAACAC  GTGCACTCATGAGGGTTTCGTA  TAGGCTGTGTAGCAGAGAGCGATGATGAAA | none  none  ATTO647N-BHQ2 | 500  600  500 |
| DETOX (C) | CYP9J28_F  CYP9J28_R  CYP9J28_P | GACAAGTACCGAGGAGTCAAAGTTT  TTAACGGCCACCTGCTTGAT  ACGTACGTCATTCGCGATCCGGA | none  none  HEX-BHQ1 | 500  600  400 |
| DETOX (C) | CYP9M6_F  CYP9M6_R  CYP9M6_P | CGTGATCTGTTTCAAAAGCTTGG  CCAACTGCTTTCCCCTTTTG  TCGGTGCACAATCCAAACAACGAGTT | none  none  ATTO647N-BHQ2 | 400  200  400 |
| DETOX (D) | CYP9J32_F  CYP9J32_R  CYP9J32_P | CTACTTCCACGACAAGCCGATAC  GTCATATCAAACAGTCCAAAAATCTTAGC  TTCCGCTCTTGGGCAGTACCGGTC | none  none  HEX-BHQ1 | 500  600  400 |

F: Foword, R: Revers, P: Pro
