# Supplementary material for: Insecticide resistance status and mechanisms in Aedes aegypti populations from Senegal
Source: PLoS Negl Trop Dis. 2021 May 10;15(5):e0009393. doi: 10.1371/journal.pntd.0009393 (PMC8136859; doi:10.1371/journal.pntd.0009393)
Supplement: S7 Table — Bold letters indicate statistical significance for all three comparisons. NO: New Orleans susceptible lab strain; LV: Liverpool black–eyed susceptible lab strain; RF: Rockefeller susceptible lab strain; CI: confidence interval. (DOCX) [file pntd.0009393.s010.docx]

**S7 Table Expression analysis of the detoxification genes analyzed in the seven resistant mosquito populations compared to three susceptible mosquito strains (New Orleans, Liverpool, and Rockefeller). Bold letters indicate statistical significance for all three comparisons.**

| **Population** | **Comparison** | **Detoxification gene fold changes** | | | | | | |
| --- | --- | --- | --- | --- | --- | --- | --- | --- |
|  |  | **(95% CI), P value** | | | | | | |
|  |  | ***CYP6BB2*** | ***CYP9J26*** | ***GSTD4*** | ***CCEae3a*** | ***CYP9J28*** | ***CYP9M6*** | ***CYP9J32*** |
| **Mbour** | *vs ΝΟ* | **39.8** (13.2–122) | **244** (78.3–1641) | **46.5** (1.13–48) | **4.67** (2.35–8.87) | **86.8** (37.5–232) | 3.01 (1.07–7.0) | **257** (69.1–785) |
|  | *P* | 0.007 | 0.005 | 0.022 | 0.008 | 0.002 | 0.032 | 0.006 |
|  | *vs LV* | **31.3** (6.65–257) | **23.3** (10.6–74.6) | **69.1** (2.53–405) | **5.92** (2.40–17.7) | **62.9** (16.4–244) | 0.376 (0.11–1.01) | **30.8** (9.01–111) |
|  | *P* | 0.012 | 0.021 | 0.016 | 0.016 | < 0.001 | 0.059 | 0.004 |
|  | *vs RF* | **17.5** (10.4–34.4) | **27.5** (14.0– 84.0) | **129.1** (73.0–193) | **1.76** (1.02–2.89) | **10.5** (6.80–18.3) | 1.34 (0.579–2.16) | **12.7** (7.08–25.4) |
|  | *P* | 0.037 | 0.04 | < 0.001 | 0.022 | 0.043 | 0.391 | < 0.001 |
| **Fatick** | *vs ΝΟ* | **34.3** (16.3–62.5) | **220** (95.7–863) | **119** (18.0–317) | **11.2** (5.93–20.5) | **38.9** (12.5–117) | 6.68 (3.83–12.5) | **88.0** (30.8–209) |
|  | *P* | 0.008 | < 0.001 | 0.007 | 0.013 | 0.002 | < 0.001 | 0.004 |
|  | *vs LV* | **27.0** (8.85–143) | **20.9** (13.2–33.1**)** | **177** (91.4–266) | **14.2** (6.07–40.8**)** | **28.2** (6.57–121) | 0.834 (0.41–1.91) | **10.6** (4.05–30.0) |
|  | *P* | 0.011 | 0.011 | 0.015 | 0.015 | < 0.001 | 0.582 | 0.001 |
|  | *vs RF* | **15.1** (14.1–16.6) | **24.7** (17.2–38.6) | **73.6** (34.9–126) | **4.54** (2.66–6.56) | **4.70** (2.28–8.91) | 2.97 (2.19–4.29) | **4.37** (3.18–7.01) |
|  | *P* | 0.031 | 0.031 | < 0.001 | < 0.001 | 0.023 | 0.035 | < 0.001 |
| **Louga** | *vs ΝΟ* | **29.0** (10.4–63.9) | **224** (72.8–1066) | **115** (13.2–473) | **8.75** (4.27–20.7) | **47.0** (18.2–127) | 8.21 (5.38–13.3) | **123** (41.1–298) |
|  | *P* | 0.007 | 0.003 | 0.006 | 0.017 | 0.003 | < 0.001 | 0.012 |
|  | *vs LV* | **22.8** (5.24–146) | **21.3** (10.0–43.3) | **170** (64.6–431) | **11.1** (4.37–38.7) | **34.0** (8.70–131) | 1.03 (0.589–1.91) | **14.7** (5.36–42.8) |
|  | *P* | 0.015 | 0.017 | 0.021 | 0.032 | < 0.001 | 0.941 | 0.001 |
|  | *vs RF* | **12.8** (8.22–17.0) | **25.2** (13.1–49.3) | **62.0** (21.3–197) | **3.65** (1.93–6.90) | **5.68** (3.32–9.63) | 3.65 (3.08–4.04) | **6.08** (4.21–10.0) |
|  | *P* | 0.047 | 0.040 | < 0.001 | 0.025 | 0.037 | 0.037 | 0.028 |
| **Touba** | *vs ΝΟ* | **38.3** (13.1–125) | **235** (70.0–1293) | **106** (7.71–1117) | **5.93** (2.89–15.9) | **25.8** (8.19–96.2) | **29.8** (13.2–65.8) | **145** (39–476) |
|  | *P* | 0.006 | < 0.001 | 0.005 | 0.010 | 0.01 | 0.011 | 0.008 |
|  | *vs LV* | **30.1** (7.00–255) | **22.3** (9.7–57.7) | **157** (17.2–1018) | **7.52** (3.05–28.7) | **18.7** (3.78–99.5) | **3.72** (1.36–9.97) | **17.5** (5.09–67.2) |
|  | *P* | 0.008 | 0.002 | 0.015 | 0.015 | 0.017 | 0.021 | 0.002 |
|  | *vs RF* | **16.9** (11.2–35.1) | **26.3** (12.6–65) | **65.6** (6.83–471) | **2.41** (1.25–5.32) | **2.88** (1.42–7.72) | **13.5** (7.31–22.4) | **7.22** (4–15.14) |
|  | *P* | 0.02 | 0.013 | < 0.001 | < 0.001 | 0.020 | 0.022 | < 0.001 |
| **Dakar** | *vs ΝΟ* | **15.63** (5.62–34.5) | **104** (42.2 – 438) | **29. 5**(2.83–176) | **5.15** (3.25–8.52) | 20.7 (6.07–71.8) | 3.77 (2.19–6.91) | **72.1** (2.91–27.6) |
|  | *P* | 0.009 | < 0.001 | 0.008 | 0.009 | 0.011 | 0.009 | 0.014 |
|  | *vs LV* | **12.3** (2.84–79.0) | **9.90** (5.84–17.4) | **43.8** (13.4–161) | **6.52** (3.49–17.0) | 15.0 (3.08–76.4) | 0.47 (0.238–1.03) | **8.65** (2.91–27.6) |
|  | *P* | 0.034 | 0.003 | 0.017 | 0.012 | 0.039 | 0.125 | 0.004 |
|  | *vs RF* | **6.88** (4.46–9.23) | **11.7**(7.59–20.0) | **18.2**(5.28–74.5) | **2.09**(1.43–2.80) | 2.50 (0.99–5.85) | 1.67 (0.909–2.31) | **3.58** (2.28–6.41) |
|  | *P* | 0.031 | 0.012 | 0.031 | 0.048 | 0.08 | 0.08 | < 0.001 |
| **Matam** | *vs ΝΟ* | **17.5** (6.15–53.6) | **90.8** (29.3–543) | **13.4** (1.84–47.8) | 2.81 (1.39–6.79) | 27.5 (4.91–117) | 5.29 (2.21–11.5) | **91.3** (30.9–222**)** |
|  | *P* | 0.004 | < 0.001 | 0.017 | 0.009 | 0.002 | < 0.001 | 0.002 |
|  | *vs LV* | **13.7** (3.31–111) | **8.64** (4.03–24.6) | **19.9** (5.98–40.1) | 3.56 (1.43–12.6) | 19.9 (3.07–124) | 0.66 (0.227–1.68) | **11** (4.03–31.8) |
|  | *P* | 0.003 | 0.003 | 0.018 | 0.040 | < 0.001 | 0.292 | 0.002 |
|  | *vs RF* | **7.71** (5.2–15.1) | **10.2** (5.27–27.8) | **8.28** (2.38–19.1) | 1.14 (0.60–2.28) | 3.32 (0.896–9.31) | 2.35 (1.20–3.69) | **4.53** (3.16–7.43) |
|  | *P* | 0.016 | 0.016 | < 0.001 | 0.662 | 0.108 | 0.049 | < 0.001 |
| **Barkedji** | *vs ΝΟ* | **28.0** (8.85–73.4) | **169** (48.2–852) | **49** (4.39–410) | 2.33 (0.89–6.34) | **86.9** (31.1–259) | 6.46 (2.70–14.7) | **200** (42.4–714) |
|  | *P* | 0.007 | < 0.001 | 0.001 | 0.018 | 0.012 | 0.010 | 0.011 |
|  | *vs LV* | **22.0** (4.47–165) | **16.1** (6.68–34.1) | **72.6** (12.3–374) | 2.96 (0.91–11.8) | **62.9** (14.6–273) | 0.80 (0.277–2.22) | **24.0** (5.53–101) |
|  | *P* | 0.007 | 0.007 | 0.006 | 0.131 | 0.020 | 0.59 | 0.002 |
|  | *vs RF* | **12.3** (7.02–20.6) | **19.0** (8.68 – 39.1) | **30.3** (4.88 – 173) | 0.947 (0.4–2.13) | **10.1** (5.40–20.6) | 2.87 (0.962–4.98) | **9.93** (4.34–22.9) |
|  | *P* | 0.037 | 0.037 | < 0.001 | 0.984 | 0.028 | 0.09 | < 0.001 |

NO: New Orleans susceptible lab strain; LV: Liverpool black–eyed susceptible lab strain; RF: Rockefeller susceptible lab strain; CI: confidence interval
